# Disorder-specific risk factors of suicidal behaviour among serving and veteran Canadian Armed Forces Members with baseline mental health diagnoses

**DOI:** 10.17269/s41997-025-01054-0

**Published:** 2025-06-12

**Authors:** Essence Perera, Tracie O. Afifi, Murray W. Enns, Natalie Mota, Jitender Sareen, Shay-Lee Bolton

**Affiliations:** 1 Department of Community Health Sciences, University of Manitoba, Winnipeg, MB Canada; 2 Department of Psychiatry, University of Manitoba, Winnipeg, MB Canada; 3 Department of Clinical Health Psychology, University of Manitoba, Winnipeg, MB Canada

**Keywords:** Mental health, Military, Veterans, Suicide, Risk factors, Protective factors, Santé mentale, Militaire, Anciens combattants, Suicide, Facteurs de risqué, Facteurs de protection

## Abstract

**Objectives:**

Many Canadian Armed Forces (CAF) members and veterans will receive a mental disorder diagnosis, and a high percentage will also experience suicidal behaviours. This study examined demographic characteristics, distal and proximal risk factors, and protective factors, and their relationship to suicidal behaviour (ideation, plans, and attempts) among CAF members and veterans who met criteria for a mental disorder at baseline.

**Methods:**

Data from the 2018 CAF Members and Veterans Mental Health Follow-up Survey (*n* = 2941) were utilized. Mental disorder diagnoses were assessed through structured diagnostic interview. Generalized linear models were conducted using subsamples of individuals with a lifetime baseline diagnosis of (a) major depressive episode (MDE), (b) posttraumatic stress disorder (PTSD), and (c) an anxiety disorder (AD; social phobia, generalized, panic).

**Results:**

Across mental disorder subsamples of those with MDE and AD, land environmental command at baseline was associated with increased prevalence of suicidal behaviour. Risk factors for suicidal behaviour across all subsamples included baseline suicidal behaviour, greater level of self-medication and avoidant coping style, greater level of baseline work stress, greater number of traumatic experiences, persistence or recurrence of index mental disorder, current comorbid mental disorder, current physical health condition, exposure to “other” traumatic experiences, and alcohol use disorder. Protective factors across all subsamples included greater level of current problem-solving coping style. Disorder-specific factors were also identified.

**Conclusion:**

This study identified characteristics of individuals living with mental disorders who might be at high risk of suicidal behaviour, highlighting potential areas for targeted interventions in this key population.

## Résumé

**Objectifs:**

Un grand nombre de membres et anciens combattants des Forces armées canadiennes (FAC) recevront un diagnostic de trouble mental, et un pourcentage élevé vivra également des comportements suicidaires. Cette étude a examiné les caractéristiques démographiques, les facteurs de risque distaux et proximaux, ainsi que les facteurs de protection, et leur relation avec les comportements suicidaires (idées, plans et tentatives) chez les membres et anciens combattants des FAC ayant reçu un diagnostic de trouble mental au départ.

**Méthodes:**

Les données proviennent de l’Enquête de suivi sur la santé mentale des membres et des anciens combattants des FAC de 2018 (*n* = 2941). Les diagnostics de troubles mentaux ont été évalués au moyen d’une entrevue diagnostique structurée. Des modèles linéaires généralisés ont été réalisés auprès de sous-échantillons de personnes ayant reçu un diagnostic à vie, au départ, de: a) épisode dépressif majeur (EDM), b) trouble de stress post-traumatique (TSPT), et c) trouble anxieux (TA; phobie sociale, trouble d’anxiété généralisée ou trouble panique).

**Résultats:**

Parmi les sous-échantillons de personnes atteintes d’un EDM ou d’un TA, le fait d’être sous le commandement terrestre au départ était associé à une prévalence accrue de comportements suicidaires. Les facteurs de risque communs à tous les sous-échantillons comprenaient: des antécédents de comportements suicidaires, un style d’adaptation basé sur l’automédication ou l’évitement, un stress professionnel antérieur plus élevé, un plus grand nombre d’expériences traumatiques, la persistance ou la récurrence du trouble mental principal, la présence actuelle d’un trouble mental concomitant, un problème de santé physique actuel, une exposition accrue à «d’autres» expériences traumatiques et un trouble lié à la consommation d’alcool. Les facteurs de protection communs à tous les sous-groupes incluaient un recours plus fréquent à un style d’adaptation basé sur la résolution de problèmes. Des facteurs propres à chaque sous-échantillon ont également été relevés.

**Conclusion:**

Cette étude a permis d’identifier les caractéristiques des personnes vivant avec un trouble mental qui pourraient être à risque élevé de comportements suicidaires, mettant en lumière des domaines potentiels d’intervention ciblée auprès de cette population clé.

## Introduction

Suicide is a crucial global public health issue and is currently the twelfth leading cause of death in Canada (Statistics Canada, 2022). There is a strong association between mental disorders and suicidal behaviour (i.e., suicidal ideation, plans, and attempts), such that many individuals with suicidal behaviour also meet criteria for a mental disorder (Moitra et al., 2021). However, a large percentage of those with mental disorders do not experience suicidal behaviour (Bachmann, 2018; Cai et al., 2021). Individuals with a mental disorder represent a group with distinct characteristics and risk factors for developing suicidal behaviour. Since it is known that mental disorders are prevalent among those who experience suicidal behaviour, it is clinically useful and relevant to identify who, among all those with a mental disorder, are most at risk of suicidal behaviour (Bachmann, 2018; Moitra et al., 2021). There is a lack of research examining the types of risk and protective factors that can impact likelihood of suicidal behaviour among military personnel and veterans with a mental disorder. It is also unclear whether these factors are consistent across a range of mental disorder presentations.

Among military personnel, demographic characteristics, such as male sex, unpartnered marital status, lower education, lower income, and land environmental command, have been associated with suicidal behaviour (Nichter et al., 2021; Nock et al., 2013). Distal risk factors, including prior suicidal behaviour, history of a mental disorder (e.g., depression, anxiety, posttraumatic stress disorder), and prior stressors or traumatic events (e.g., child maltreatment, deployment traumatic experiences), have also shown a relationship in previous work (Afifi et al., 2016; Bryan et al., 2018; Nichter et al., 2021). Proximal risk factors, including stressors (e.g., transitioning to civilian life and leaving military service), recent traumatic experiences (e.g., witnessing death, military sexual trauma), persistence of a mental disorder, physical and chronic pain conditions, alcohol use disorders, and maladaptive coping styles (e.g., using illicit substances, emotional avoidance), have similarly been linked (Ravindran et al., 2020; Racine, 2018; Werbart Tornblom et al., 2021). Overall, evidence suggests that a higher number of stressors and traumatic exposures is indicative of greater suicidal risk (Monteith et al., 2018). Although limited research exists related to protective factors in the context of suicidal behaviour, extant research suggests that social support and use of adaptive coping styles may be protective (Holman & Williams, 2022). Existing research has not extensively explored variations in demographics, risk, and protective factors based on mental disorder diagnoses; however, gaining a deeper understanding of these characteristics and their links to suicidal behaviour can enable health professionals to develop targeted, tailored interventions for individuals living with mental disorders.

Despite literature highlighting the elevated risk for suicidal behaviour among individuals with a mental disorder, a dearth of research exists as to whether there are certain characteristics that impact this risk among military personnel and veterans (Moitra et al., 2021). Research to date has not examined differences in risk factors associated with suicidal behaviour by specific mental disorder diagnoses. A better understanding of these factors (i.e., demographics, risk, protective) for members and veterans is needed to provide clarity on risk for suicidal behaviour. To address this gap in the literature, this study aimed to examine (a) demographic characteristics, (b) risk factors, and (c) protective factors associated with increased or decreased prevalence of suicidal behaviour across different mental disorders diagnosed at baseline (i.e., major depressive episode, posttraumatic stress disorder, anxiety disorder) over a 16-year follow-up period among CAF members and veterans.

## Methods

### Data and sample

This study utilized data from the Canadian Armed Forces Members and Veterans Mental Health Follow-up Survey (CAFVMHS). The CAFVMHS, collected in 2018 through a collaboration between Statistics Canada and the Department of National Defence, follows a representative sample of 5155 CAF personnel from the 2002 Canadian Community Health Survey–Canadian Forces Supplement. Of those eligible for re-interview in 2018, a total of 2941 participated in the follow-up survey (further information on survey methods have been previously published; Afifi et al., 2021a).

### Subsample creation

The World Health Organization – Composite International Diagnostic Interview (WHO-CIDI), a reliable and valid structured diagnostic interview, was used by trained lay interviewers to assess mental disorders in 2002 and 2018 according to the Diagnostic and Statistical Manual of Mental Disorders (DSM)−4th Edition (Kessler et al., 2004). In 2002, the following lifetime diagnoses were assessed: major depressive episode (MDE), posttraumatic stress disorder (PTSD), generalized anxiety disorder (GAD), panic disorder (PD), and social phobia (SP). A variable of “any anxiety disorder” was created based on endorsement of any one of the following anxiety disorders: PD, GAD, and/or SP; this approach has been used in prior work with this dataset (Pankratz et al., 2022). Analyses were conducted in the following three subsamples of participants at baseline: those who met criteria for (A) MDE, those for (B) PTSD, and those for (C) one or more anxiety disorder(s) (AD). A participant may have been included in multiple subsamples if they had more than one mental disorder at baseline (e.g., both 2002 lifetime MDE and PTSD).

### Measures

#### Suicidal behaviour

Lifetime suicidal behaviour was assessed in 2002 with separate questions regarding suicidal ideation, plans, and attempts. An integrated variable of “suicidal behaviour” (i.e., ideation, plans, or attempts) between 2002 and 2018 was used as the main outcome. A single variable of baseline suicidal behaviour was created based on the response of “yes” to suicidal behaviour in 2002. In 2018, suicidal behaviours were assessed with similar questions as in 2002.

#### Demographic characteristics

Demographic characteristics assessed at baseline (i.e., in 2002) included age (continuous, in years), sex (male [reference] vs. female), marital status (partnered: married or common law [reference] vs. not partnered: separated, divorced, or widowed, never married), level of educational attainment (high school or less [reference] vs. secondary school or higher), and household income dichotomized based on the median Canadian income in 2002 ($0‒$49,999 [reference] vs. $50,000 +). Military characteristics examined included environmental command (land [reference]/army, air, sea) and military rank (junior non-commissioned member [reference] and senior non-commissioned member vs. commissioned officer). Also, 2002 lifetime deployment status was examined (never deployed [reference] vs. deployed at least once).

#### Traumatic experiences

In 2002 and 2018, respondents were asked about lifetime exposure to 28 potentially traumatic events as part of the WHO-CIDI PTSD module (Kessler et al., 2004). Events significantly associated with deployment formed a “deployment-associated traumatic experiences” variable (12 items, e.g., combat, seen atrocities or massacres), and remaining events were collapsed into “sexual traumatic experiences” (2 items, e.g., unwanted touching; sexual assault) or “other traumatic experiences” (14 items, e.g., harm to a loved one; injured, tortured, or killed another person; categorization based on prior work; Enns et al., 2021). Two events including witnessing domestic violence in childhood and childhood abuse were examined in 2002 but were excluded in 2018. Additionally, a count of the total number of traumatic events at baseline (in 2002) and follow-up (in 2018) were created and examined.

#### Work stress

Serving CAF members or those currently employed were asked about work stress using a 12-item measure based on the Job Content Questionnaire in 2002 and 2018 (Karasek et al., 1998). Items were assessed on a 5-point ordinal scale from strongly agree to strongly disagree and summed to create a total score ranging from 0 to 40. A continuous level of work stress was created and examined at baseline (in 2002) and follow-up (in 2018).

#### Deployment-related experiences (DEX)

Eight items (no/yes) were used to assess lifetime exposures that occurred during CAF deployments as of 2018 (e.g., ever felt responsible for the death of a Canadian or ally personnel; Boulos & Fikretoglu, 2018). Number of DEX was created and examined based on whether the respondent reported exposure to one or more DEX items assessed in the survey.

#### Physical health conditions

The CAFVMHS assessed 19 current physical health conditions, diagnosed by a health professional lasting 6 months or more (e.g., asthma, diabetes, traumatic brain injury effects; a complete list of disorders has been published; Afifi et al., 2021a). A variable (no [reference]/yes) of any condition known to be associated with chronic pain was created based on endorsement of any one of the following: arthritis, back problems, migraines, and gastrointestinal conditions based on prior work with this dataset (Perera et al., 2021). Additionally, a “chronic physical health condition” variable captured respondents who reported any other physical health condition not related to chronic pain.

#### Childhood maltreatment

In 2018, childhood maltreatment history was assessed and included items that occurred prior to 16 years of age: sexual abuse, physical abuse, emotional abuse, exposure to intimate partner violence (categorizations based on prior work; Afifi et al., 2021b). Also, neglect was assessed using a single item asking whether the respondent was left unsupervised before age 10. A “childhood maltreatment” variable (no [reference]/yes) was created based on whether respondents endorsed any abuse or neglect.

#### Release from service

Respondents were asked whether they were currently actively serving as a CAF member or whether they had released from service (i.e., became a veteran). A variable (no[reference]/yes) was created to indicate those who were a veteran in 2018.

#### Current difficulty sleeping

In 2018, respondents rated sleep difficulties from 1 (“none of the time”) to 5 (“all of the time”). This was categorized into low (reference) and high difficulty based on a median split, with those at or below the median classified as “low” difficulty and those above the median as “high” difficulty.

#### Social support

At both baseline (in 2002) and follow-up (in 2018), social support was assessed using the Medical Outcomes Study Social Support Survey (Sherbourne & Stewart, 1991). This 19-item measure assessed social support with total scores ranging from 0 to 76, where lower scores represent lower support. Level of social support was examined as a continuous variable at baseline and follow-up.

#### Coping with stress

At both baseline (in 2002) and follow-up (in 2018), respondents were asked 14 questions about coping strategies derived from the Ways of Coping Questionnaire, the COPE Scale, and the Coping Strategy Indicator (Amirkhan, 1990; Carver et al., 1989; Folkman & Lazarus, 1985). Items were assessed on a scale from 1 to 4 (often to never). Previous factor analysis resulted in three distinct styles: avoidant, problem-focused, and self-medication (Mota et al., 2013). Coping styles were not mutually exclusive and were examined separately as continuous variables to the degree that each was used at baseline and follow-up.

#### Comorbid mental disorders

As stated, mental disorders were assessed by the WHO-CIDI using trained lay interviewers (Kessler et al., 2004). Comorbid mental disorders were assessed based on the endorsement of “yes” to any other DSM-IV mental disorder, excluding the one used for subsample stratification at baseline. For example, if data were stratified by those who had MDE, then a diagnosis of any one of the other disorders (e.g., PTSD or AD) would be used to indicate comorbidity. Comorbid alcohol dependence was also captured by the WHO-CIDI and was assessed only in 2018 (no [reference]/yes). A “persistence or recurrence” variable was created for each mental disorder, indicating individuals who met criteria for the same lifetime disorder in both 2002 and between 2002 and 2018 (no [reference]/yes).

### Data analysis

Data were analyzed in STATA (16.1, StataCorp LLC, College Station, TX). Sampling and bootstrapping weights created by Statistics Canada were used to ensure representativeness of the 2018 sample to the CAF Regular Force population in 2002 and to account for nonresponse. Analyses were conducted separately for each of the three disorder subsamples. Descriptive statistics were computed to examine the prevalence of suicidal behaviour and of demographic predictors, as well as the prevalence of risk and protective factors by subsample. Further, generalized linear models with a log-binomial link function were used to identify which factors were significantly associated with higher or lower prevalence of suicidal behaviour by disorder subsample. Forest plots of prevalence ratios and corresponding 95% confidence intervals (CI) were generated in R (version 4.3.2) using the ggplot2 and dplyr packages.

## Results

The prevalence of suicidal behaviour at follow-up among individuals with baseline MDE, PTSD, or AD was 38.2%, 40.2%, and 38.2%, respectively. Table 1 displays the demographic characteristics of individuals who experienced suicidal behaviour at follow-up by mental disorder subsample. The baseline mean age of individuals with mental disorders who later experienced suicidal behaviour was approximately 35 to 36 years.

**Table 1:** Baseline demographic predictors of suicidal behaviour among individuals with mental disorders

Baseline characteristics	Mental disorder subsample		
	MDE and SB	PTSD and SB	AD and SB
	Mean (SE)	Mean (SE)	Mean (SE)
Age	35.35 (0.59)	36.20 (0.89)	35.54 (0.62)
	**%**	**%**	**%**
Sex			
Male	81.3^a^	76.3	86.5
Female	18.7	23.7	13.5
Marital status			
Partnered	62.6	54.8	71.8
Not partnered	37.4	45.2	28.2
Education			
High school graduate or less	46.1	53.4	50.5
At least some post-secondary	53.9	46.6	49.5
Household income			
$0‒$49,999	23.9	22.1	20.4
$50,000 +	76.1	77.9	79.6
Environmental command			
Land	61.3	68.8	59.3
Air	22.9	17.1	21.6
Sea	15.8	14.1	19.0
Rank			
Junior and Senior NCM	86.5	90.5	93.3
Officer	13.5	9.5	6.7
Deployment status			
Never deployed	40.5	28.1	41.7
Prior deployment	59.5	72.0	58.3

Percentages (%) are weighted. ^a^Percentage indicates that, among those with MDE, 81.3% of males experienced suicidal behaviour at follow-up. Partnered marital status included those who were married or common-law, and not partnered marital status included those who were widowed, separated, divorced, or single/never married. *SB* suicidal behaviour between 2002 and 2018, *MDE* major depressive episode, *PTSD* posttraumatic stress disorder, *AD* anxiety disorder, *NCM* non-commissioned member, *SE* standard error

Tables 2 and 3 present the prevalence and means of baseline risk factors for later suicidal behaviour among individuals in each subsample. Approximately 59‒69% of individuals with a mental disorder and future suicidal behaviour had a baseline comorbid disorder. For self-medication coping styles, mean values were similar across mental disorder subsamples and suicidal behaviour at follow-up, with a mean of 4.86 (standard error; SE = 0.17) for MDE, a mean of 4.94 (SE = 0.23) for PTSD, and a mean of 4.86 (SE = 0.17) for AD. Level of social support was slightly higher among individuals with AD and suicidal behaviour at follow-up (*M* = 59.19, SE = 1.54), while the MDE and PTSD subsamples had means of 58.79 (SE = 1.36) and 57.99 (SE = 1.97), respectively.

**Table 2:** Frequency of baseline dichotomous risk factors for suicidal behaviour among individuals with mental disorders

Baseline factors (2002)	Mental disorder subsample		
	MDE and SB	PTSD and SB	AD and SB
	%	%	%
Suicidal behaviour			
No	44.7	46.6	33.0
Yes	55.3^a^	53.4	67.0
Comorbid mental disorder			
No	41.3	30.9	40.1
Yes	58.8	69.1	59.9
Deployment-associated traumatic experiences			
No	11.8	8.2	17.0
Yes	88.2	91.8	83.0
Sexual traumatic experiences			
No	74.1	74.1	75.1
Yes	25.9	25.9	24.9
“Other” traumatic experiences			
No	17.9	8.7	8.7
Yes	82.1	91.3	91.3
Childhood maltreatment			
No	15.3	21.2	20.2
Yes	84.7	78.8	79.8

*SB* suicidal behaviour between 2002 and 2018, *MDE* major depressive episode, *PTSD* posttraumatic stress disorder, *AD* anxiety disorder

Percentages (%) are weighted. ^a^Percentages are to be interpreted as, for example among those with MDE, 55.3% of those with baseline suicidal behaviour experienced suicidal behaviour at follow-up

**Table 3:** Descriptives of baseline continuous risk and protective factors of suicidal behaviour among individuals with mental disorders

Baseline factors (2002)	Mental disorder subsample		
	MDE and SB	PTSD and SB	AD and SB
	Mean (SE)	Mean (SE)	Mean (SE)
Level of self-medication coping	4.86 (0.17)	4.94 (0.23)	4.86 (0.17)
Level of avoidant coping	12.97 (0.28)	13.16 (0.40)	13.33 (0.28)
Level of work stress	20.93 (0.55)	21.44 (0.67)	20.89 (0.58)
Number of traumatic experiences	5.34 (0.26)	7.02 (0.43)	5.37 (0.35)
Level of problem-solving coping	13.19 (0.17)	13.25 (0.19)	12.76 (0.21)
Level of social support	58.79 (1.36)	57.99 (1.97)	59.19 (1.54)

*SB* suicidal behaviour between 2002 and 2018, *MDE* major depressive episode, *PTSD* posttraumatic stress disorder, *AD* anxiety disorder, *SE* standard error

Tables 4 and 5 display descriptives of current correlates of suicidal behaviour among individuals with mental disorders. Among those with a mental disorder, about 27‒32% of those with an alcohol use disorder experienced suicidal behaviour at follow-up. Additionally, the number of traumatic experiences was highest in the PTSD subsample (*M* = 4.45, SE = 0.55), while MDE and AD subsamples had means of *M* = 3.89, SE = 0.36, and *M* = 3.32, SE = 0.35, respectively. For problem-solving coping styles, mean values were similar across mental disorder subsamples who reported suicidal behaviour at follow-up, with means of 12.46 (SE = 0.18) for MDE, 12.19 (SE = 0.26) for PTSD, and 12.12 (SE = 0.21) for AD.

**Table 4:** Frequency of dichotomous correlates of suicidal behaviour among individuals with mental disorders

Current factors (2002‒2018)	Mental disorder subsample		
	MDE and SB	PTSD and SB	AD and SB
	%	%	%
Persistence or recurrence of index mental disorder			
No	14.3	31.7	22.5
Yes	85.7^a^	68.3	77.5
Comorbid mental disorder			
No	49.6	~	19.2
Yes	80.4	~	80.9
Alcohol use disorder			
No	73.0	68.4	71.2
Yes	27.0	31.6	28.8
Chronic physical health condition (except chronic pain)			
No	8.7	8.8	12.4
Yes	91.3	91.2	87.6
Chronic pain condition			
No	20.7	18.7	25.8
Yes	79.3	81.3	74.2
Difficulty sleeping			
Low	43.2	50.7	45.3
High	56.9	49.3	54.7
Deployment-associated traumatic experiences			
No	34.1	33.1	40.5
Yes	65.9	67.0	59.5
Sexual traumatic experiences			
No	92.8	88.3	92.2
Yes	7.2	11.7	7.8
“Other” traumatic experiences			
No	30.4	26.2	36.1
Yes	69.6	73.9	63.9
Released from service			
No	23.3	14.9	19.1
Yes	76.7	85.1	80.9

Percentages (%) are weighted. ^a^Percentages are to be interpreted as, among those with MDE, 85.7% of those with a persistent or recurring mental disorder experienced suicidal behaviour at follow-up

*SB* suicidal behaviour between 2002 and 2018, *MDE* major depressive episode, *PTSD* posttraumatic stress disorder, *AD* anxiety disorder

The cells denoted with “ ~ ” had cell size issues and were therefore not able to be released by Statistics Canada regulations

**Table 5:** Descriptives of continuous current correlates of suicidal behaviour among individuals with mental disorders

Current factors (2002‒2018)	Mental disorder subsample		
	MDE and SB	PTSD and SB	AD and SB
	Mean (SE)	Mean (SE)	Mean (SE)
Level of self-medication coping	7.33 (0.16)	7.55 (0.23)	7.77 (0.17)
Level of avoidant coping	14.67 (0.27)	14.78 (0.36)	15.09 (0.27)
Level of work stress^a^	17.68 (0.66)	16.98 (0.97)	19.13 (0.90)
Number of traumatic experiences	3.89 (0.36)	4.45 (0.55)	3.32 (0.35)
Number of deployment-related experiences (DEX)	1.68 (0.20)	1.74 (0.31)	1.23 (0.19)
Level of problem-solving coping	12.46 (0.18)	12.19 (0.26)	12.12 (0.21)
Level of social support	31.85 (0.89)	32.26 (1.27)	30.62 (1.18)

^a^Among those who were currently employed

*SB* suicidal behaviour between 2002 and 2018, *MDE* major depressive episode, *PTSD* posttraumatic stress disorder, *AD* anxiety disorder, *SE* standard error

**Fig. 1:**
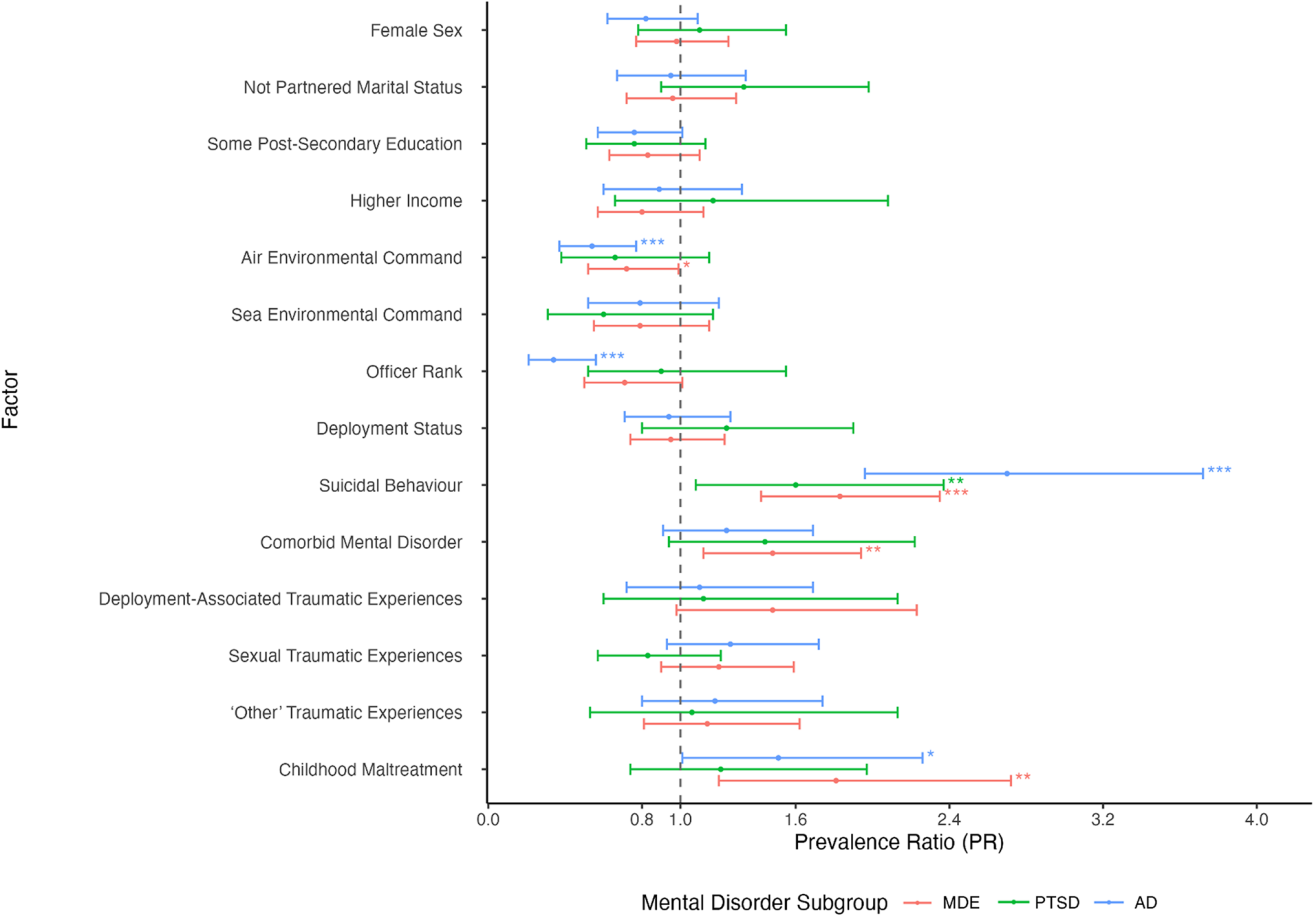
Forest plot of prevalence ratios (PRs) and 95% confidence intervals (CI) for baseline demographic and predictive factors (nominal variables) of suicidal behaviour at follow-up among CAF members and veterans with mental disorders. Dot represents PR estimate. 95% CI represented by error bars. Values to the left of 1 indicate factors associated with decreased prevalence of suicidal behaviour, while values to the right of 1 indicate increased prevalence. *MDE* major depressive episode, *PTSD* posttraumatic stress disorder, *AD* anxiety disorder. *p ≤ 0.05; **p ≤ 0.01; ***p ≤ 0.001

**Fig. 2:**
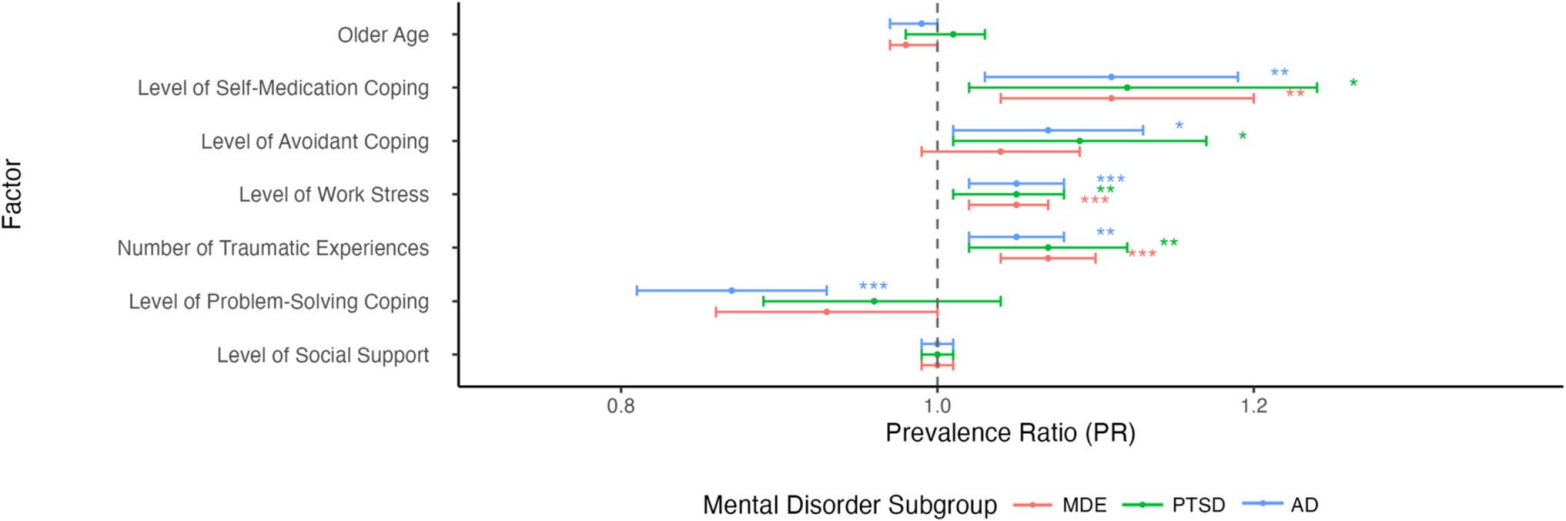
Forest plot of prevalence ratios (PRs) and 95% confidence intervals (CI) of baseline demographic and predictive factors (continuous variables) of suicidal behaviour at follow-up among CAF members and veterans with mental disorders. Dot represents PR estimate. 95% CI represented by error bars. Values to the left of 1 indicate factors associated with decreased prevalence of suicidal behaviour, while values to the right of 1 indicate increased prevalence. *MDE* major depressive episode, *PTSD* posttraumatic stress disorder, *AD* anxiety disorder. *p ≤ 0.05; **p ≤ 0.01; ***p ≤ 0.001

Figures 1 and 2 demonstrate forest plots of prevalence ratios (PRs) for baseline demographics and predictors of suicidal behaviour among subsamples of individuals with mental disorders. Among individuals with MDE and AD, air environmental command was associated with a lower prevalence of suicidal behaviour (PR = 0.54 and 0.72, respectively). Additionally, officer rank was associated with a decreased prevalence of suicidal behaviour among those with AD (PR = 0.34, 95% CI, 0.21‒0.56, *p* ≤ 0.001). Across disorders, greater level of self-medication coping, greater levels of work stress, and a greater number of traumatic experiences were associated with a higher prevalence of suicidal behaviour (PRs ranging between 1.05 and 1.07).

**Fig. 3:**
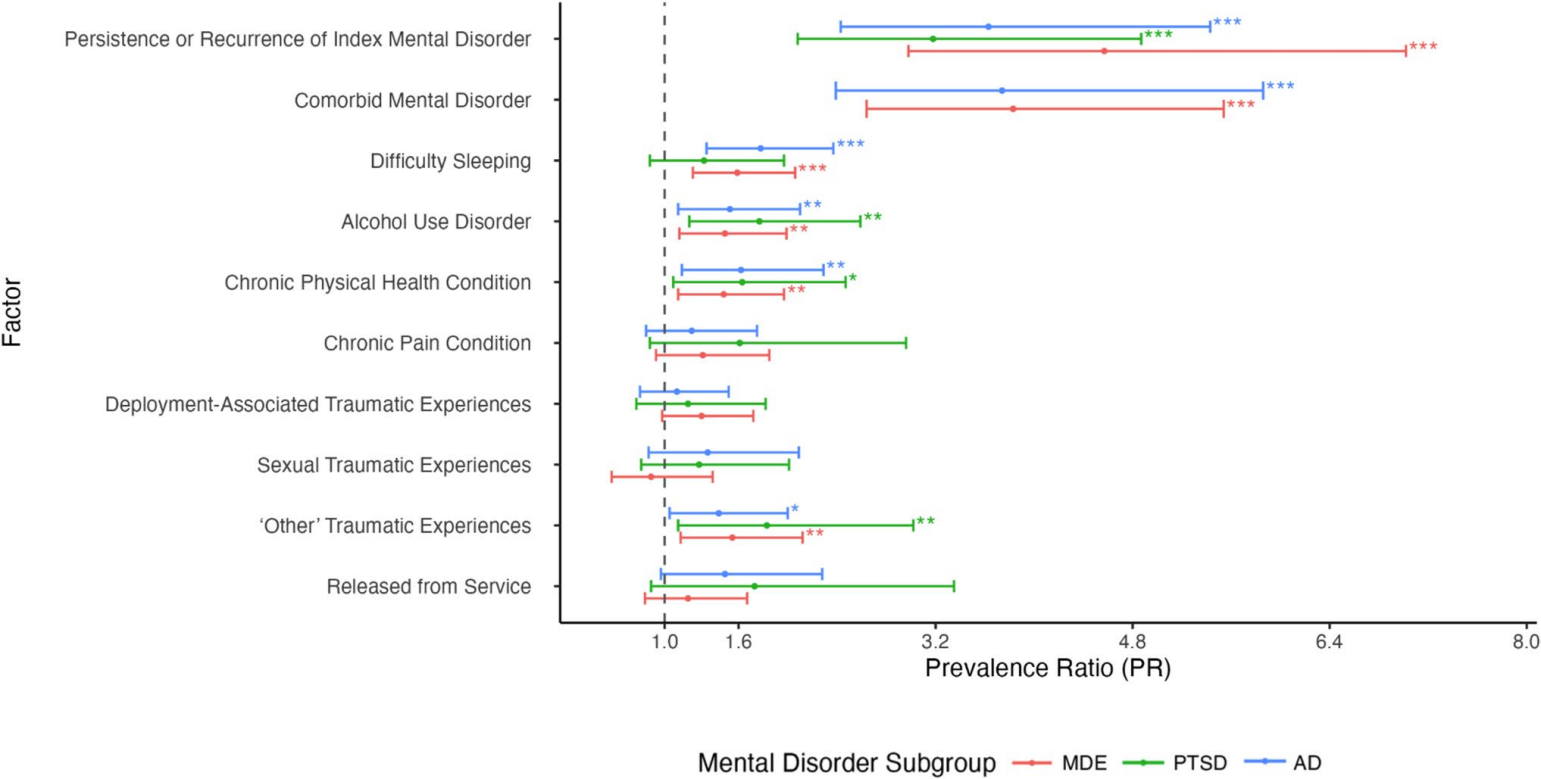
Forest plot of prevalence ratios (PRs) and 95% confidence intervals (CI) of current correlates (nominal variables) of suicidal behaviour at follow-up among CAF members and veterans with mental disorders. Dot represents PR estimate. 95% CI represented by error bars. Values to the left of 1 indicate factors associated with decreased prevalence of suicidal behaviour, while values to the right of 1 indicate increased prevalence. Cell size was too small to examine comorbid mental disorder for the PTSD subsample, though there was a trend in increased prevalence of suicidal behaviour among those who had a current comorbid mental disorder. *MDE* major depressive episode, *PTSD* posttraumatic stress disorder, *AD* anxiety disorder. *p ≤ 0.05; **p ≤ 0.01; ***p ≤ 0.001

**Fig. 4:**
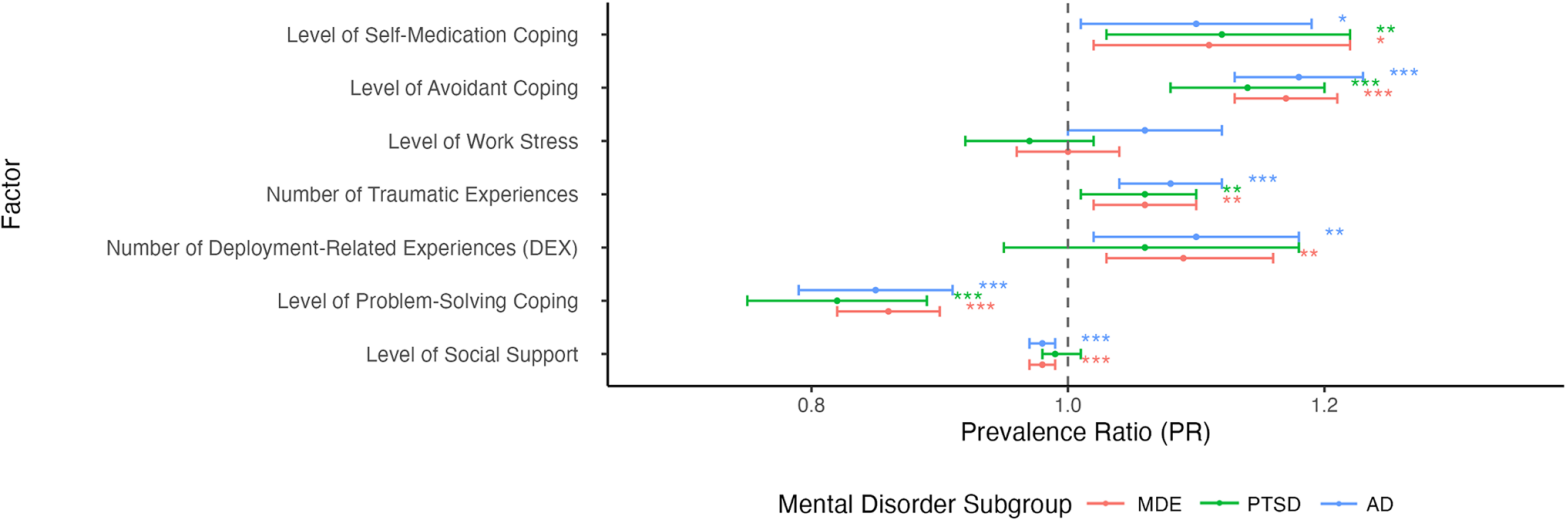
Forest plot of prevalence ratios (PRs) and 95% confidence intervals (CI) of current correlates (continuous variables) of suicidal behaviour at follow-up among CAF members and veterans with mental disorders. Dot represents PR estimate. 95% CI represented by error bars. Values to the left of 1 indicate factors associated with decreased prevalence of suicidal behaviour, while values to the right of 1 indicate increased prevalence. *MDE* major depressive episode, *PTSD* posttraumatic stress disorder, *AD* anxiety disorder. *p ≤ 0.05; **p ≤ 0.01; ***p ≤ 0.001

Figures 3 and 4 portray PRs for current correlates of suicidal behaviour among CAF members and veterans with mental disorders. Across all subsamples, those who had persistence or recurrence of the index mental disorder, a current alcohol use disorder, a current chronic physical health condition, greater level of self-medication and avoidant coping styles, greater number of current traumatic experiences, and exposure to “other” traumatic experiences had a higher prevalence of suicidal behaviour (PRs ranging from 1.06 to 4.57). Among those with MDE and AD, a high level of sleep difficulty was associated with a greater prevalence of suicidal behaviour (PR = 1.59 and 1.78, respectively). Greater use of current problem-solving coping was linked to a lower prevalence of suicidal behaviour for all subsamples, with PRs ranging from 0.82 to 0.86.

## Discussion

This study is the first Canadian study to shed light on the complex factors influencing suicidal behaviour among CAF members and veterans living with MDE, PTSD, and AD. By identifying specific characteristics, risk, and protective factors by mental disorder diagnosis, this study serves to inform policymakers, healthcare providers, and supports for CAF members and veterans living with mental disorders. Notably, this study found that the prevalence of suicidal behaviour among individuals with MDE, PTSD, and AD was higher than that found in previous research with general population samples, with estimates of 38.2%, 40.2%, and 38.2%, respectively (Cai et al., 2021). These results underscore the need for targeted treatments and comprehensive mental health services to address this pressing public health issue.

The current study’s findings highlight that individuals with MDE and AD in the land environmental command at baseline showed an increased prevalence of suicidal behaviour. This association is consistent with previous findings (Nock et al., 2013) and may be that individuals in the land command may be exposed to higher rates of traumatic experiences (e.g., combat operations; Boulos, 2020). Similarly, individuals with AD in junior and senior non-commissioned ranks demonstrated a higher prevalence of suicidal behaviour. Military personnel in lower ranks may be more exposed to certain work-related duties, increasing their exposure to traumatic experiences and potentially contributing to elevated suicide risk (Ursano et al., 2020). These results are in line with previous findings linking lower military rank to increased risk of suicidal behaviour (Adams et al., 2022). Among individuals with PTSD, none of the examined demographic characteristics were associated with suicidal behaviour. This contrast with findings for MDE and AD may reflect differences inherent to the disorders themselves. In PTSD, re-experiencing traumatic events and distressing symptoms may drive suicidal risk, overshadowing demographic factors. Prior research has consistently shown a strong link between PTSD and suicidal behaviour, particularly in military populations (Bachmann, 2018; Moitra et al., 2021; Nichter et al., 2021; Nock et al., 2013).

Although there were differences in which risk factors were significantly related to suicidal behaviour by disorder, baseline suicidal behaviour and greater work stress at baseline were consistently associated with increased suicidal behaviour. These findings underscore the importance of screening across presentations for specific predictors (e.g., prior suicidal behaviour). Across all three subsamples of those currently employed, greater work stress was associated with a 5% increase in prevalence of suicidal behaviour. This is in line with previous research which suggests that stressors related to the military members’ current work, whether in active service or post-service, can significantly impact the individual and can lead to increased suicidal risk (Bryan et al., 2018; Nichter et al., 2021; Nock et al., 2013). It is crucial to note that these findings highlight the importance of addressing work-related stress to promote mental health and well-being among both military personnel and veterans.

Across all subsamples, risk factors at follow-up associated with increased prevalence of suicidal behaviour included persistence or recurrence of the index mental disorder, current alcohol use disorder, current physical health conditions, exposure to “other” traumatic experiences, greater use of self-medication and avoidant coping, and greater number of traumatic experiences. Additionally, a three- to five-fold increase in the prevalence of suicidal behaviour was linked to persistence or recurrence of the index mental disorder. This highlights a significant differential relationship among individuals who did not have persistence or recurrence of the index mental disorder over the follow-up. This association showed the highest magnitude compared to all other risk factors examined, consistent with previous research suggesting suicidal behaviours are primarily influenced by factors occurring closer in time to suicidal behaviour (Werbart Tornblom et al., 2021). This finding emphasizes the importance of early and sustained treatment in mental health care. By addressing primary mental disorders promptly and effectively, healthcare providers can potentially mitigate the heightened risk of suicidal behaviour.

### Strengths and limitations

This study’s strengths include its use of secondary data with a high response rate. The large, representative sample of CAF members and veterans was followed longitudinally, allowing for a unique opportunity to assess factors influencing suicidal risk over time and across the transition from service. Several limitations must be also considered while interpreting the findings of this study. First, mental disorders were obtained through structured clinical interviews by trained lay interviewers, which may be different when compared to clinician-based diagnoses. Second, important data regarding temporality of suicidal behaviours and risk factors assessed in 2018 could not be determined as it was not assessed in the CAFVMHS. Finally, due to the cross-sectional nature of the CAFVMHS data, causal inferences cannot be made and the direction of some associations remains uncertain.

## Conclusion

Findings from this study serve to better understand trends over time of characteristics, risk, and protective factors for suicidal behaviour among CAF members with a mental disorder. For some, it captures the transition period from actively serving to veteran status. This information is clinically useful given that an individual may have already screened positive for a mental disorder by the time that they are brought to the attention of healthcare providers. Clinicians working with members and veterans living with mental disorders can use this information to enhance protective factors, such as social support and problem-solving coping skills. Findings can also be impactful in terms of screening for key risk factors, including land environment, lower rank, and history of suicidal behaviours. Moreover, our results underscore the significance of mitigating modifiable risk factors, such as self-medication and avoidant coping mechanisms. Clinicians can work to address risk factors and promote protective factors among individuals living with mental disorders, assisting them to better cope with life events and stressors and thereby reduce the risk of suicidal behaviour. Future longitudinal studies are needed to investigate the intricate relationship between the timing of experiences (e.g., onset of high work stress, traumatic events) and suicidal outcomes, providing critical insights for prevention and intervention efforts. Additionally, future research should employ larger samples to conduct further analyses of suicidal behaviours, differentiating between suicidal ideation, plans, and attempts. Given the high prevalence of mental disorders and presence of suicidal behaviours in CAF members and veterans, further research is crucial to optimize services.

## Contributions to knowledge

What does this study add to existing knowledge?

- This study is the first in Canada to explore the multifaceted factors contributing to suicidal behaviour among CAF members and veterans by mental disorder diagnosis.

What are the key implications for public health interventions, practice, or policy?

- This study highlights key screening factors (e.g., prior suicidal behaviour, greater number of deployment-related experiences, childhood maltreatment) and modifiable risk factors (e.g., self-medication and avoidant coping styles) in efforts to reduce the prevalence of suicidal behaviours for individuals working with military members and veterans who are living with a mental disorder.

## Data Availability

The dataset was available to researchers through the Research Data Centre. Researchers can access the dataset with the appropriate clearance qualifications as classified by Statistics Canada and study project approval from the Research Data Centre.

## Code availability

STATA coding files can be made available upon reasonable request.
